# Short Single-Lead ECG Signal Delineation-Based Deep Learning: Implementation in Automatic Atrial Fibrillation Identification

**DOI:** 10.3390/s22062329

**Published:** 2022-03-17

**Authors:** Bambang Tutuko, Muhammad Naufal Rachmatullah, Annisa Darmawahyuni, Siti Nurmaini, Alexander Edo Tondas, Rossi Passarella, Radiyati Umi Partan, Ahmad Rifai, Ade Iriani Sapitri, Firdaus Firdaus

**Affiliations:** 1 Intelligent System Research Group, Faculty of Computer Science, Universitas Sriwijaya, Palembang 30139, Indonesia; bambangtutuko60@gmail.com (B.T.); naufalrachmatullah@gmail.com (M.N.R.); riset.annisadarmawahyuni@gmail.com (A.D.); passarella.rossi@unsri.ac.id (R.P.); ahmadrifai@ilkom.unsri.ac.id (A.R.); adeirianisapitri13@gmail.com (A.I.S.); virdauz@gmail.com (F.F.); 2 Department of Cardiology & Vascular Medicine, Dr. Mohammad Hoesin Hospital, Palembang 30126, Indonesia; tondas2000@gmail.com; 3 Faculty of Medicine, Universitas Sriwijaya, Palembang 30139, Indonesia; radiyati.u.p@fk.unsri.ac.id

**Keywords:** delineation, electrocardiogram, convolutional neural network, long-short term memory

## Abstract

Physicians manually interpret an electrocardiogram (ECG) signal morphology in routine clinical practice. This activity is a monotonous and abstract task that relies on the experience of understanding ECG waveform meaning, including P-wave, QRS-complex, and T-wave. Such a manual process depends on signal quality and the number of leads. ECG signal classification based on deep learning (DL) has produced an automatic interpretation; however, the proposed method is used for specific abnormality conditions. When the ECG signal morphology change to other abnormalities, it cannot proceed automatically. To generalize the automatic interpretation, we aim to delineate ECG waveform. However, the output of delineation process only ECG waveform duration classes for P-wave, QRS-complex, and T-wave. It should be combined with a medical knowledge rule to produce the abnormality interpretation. The proposed model is applied for atrial fibrillation (AF) identification. This study meets the AF criteria with RR irregularities and the absence of P-waves in essential oscillations for even more accurate identification. The QT database by Physionet is utilized for developing the delineation model, and it validates with The Lobachevsky University Database. The results show that our delineation model works properly, with 98.91% sensitivity, 99.01% precision, 99.79% specificity, 99.79% accuracy, and a 98.96% F1 score. We use about 4058 normal sinus rhythm records and 1804 AF records from the experiment to identify AF conditions that are taken from three datasets. The comprehensive testing has produced higher negative predictive value and positive predictive value. This means that the proposed model can identify AF conditions from ECG signal delineation. Our approach can considerably contribute to AF diagnosis with these results.

## 1. Introduction

Electrocardiogram (ECG) is the gold standard for signal detection and an effective way to evaluate the heart’s rhythm due to its versatility and affordability as a rapid and straightforward test, even in places with scarce resources [1,2,3]. The test results obtained from an ECG device are heart rhythms, which can reveal an overview of the heart’s morphology, such as its structural and physiological conditions [3,4]. A short single-lead ECG is used for essential heart rhythms monitoring, which has a short or minimum duration (seconds) in ECG recording. Additionally, it presents only in one lead ECG signal. It is different to a standard long 12-lead ECG, which provides a complete picture of heart activity for making clinical decisions [2]. Long ECG tends to need several rounds of recording (minutes to hours) to monitor the morphology. However, health care systems emphasize simple, cost-effective solutions, scalable with automated analysis [5,6]. Several simple, low-cost, wireless wearable ECG mobile devices with a short single-lead analysis have made private screening indispensable [5]. Unfortunately, it is difficult to perform rapid and accurate diagnosis by using such devices, caused by inconsistent findings or low-wave amplitude. About 27% uninterpretable intervals due to single-lead ECG have low quality, noise, low amplitude, complexity, and non-linear characteristics [5,7]. Hence, deep investigation into ECG signal processing is needed to improve the prediction with a short single-lead device.

The morphology of a normal ECG signal indicates a P-wave, the QRS complex, and a T-wave in the interval of one heartbeat [4]. A normal ECG signal without irregular fluctuations signified a healthy patient. This is different to several heart abnormalities which can be detected by recognizing and analyzing these irregularity characteristics, such as long QT syndrome, which is characterized by a QT interval [5], and atrial fibrillation (AF) with missing P-wave [6]. The human interpretation about AF condition from ECG recordings varies widely, although the acquisition has been standardized. This is due to differences in the interpreter’s experience and expertise. In addition, human errors and inaccurate interpretations can occur [7,8]. General clinicians missed 20% of AF cases (20 of 99) on a 12-lead ECG and misinterpreted normal sinus rhythm (NSR) as AF by 8% (114 of 1355) when compared to the cardiologist reference standard [9]. The use of single-lead ECGs can increase sensitivity with interpretive software approaches and general clinicians’ interpretations [10]. While ECG signals and patterns are mainly unrecognizable by human interpreters, artificial intelligence (AI) plays a role in precision, making the ECG a robust non-invasive biomarker [11].

In the future, the amount of data generated by the ECG device to be analyzed and diagnosed will increase with the development of sensitivity sensors and other supporting devices, such as cell phones, smartwatches, and low-cost mobile ECG devices [12,13]. Therefore, using AI technology in short single-lead ECG devices will improve quality detection and reduce the burden of analyzing cardiac data. AF condition can be diagnosed using an ECG device that is identified through electrical activity during the repolarization and depolarization process. In this study, such process is represented by segmenting the duration of ECG signal waveforms (P-wave, QRS complex, T-wave) named delineation [14]. Proper and correct delineation model for ECG waveform can produce accurate diagnostics [8,15].

Currently, the AI approach with deep learning (DL) algorithms, such as deep neural networks (DNNs) [11,12] convolutional neural networks (CNNs) [13], and recurrent neural networks (RNNs) [15,16], has revolutionized the way machines interpret ECG signals. RNNs with long short-term memory (LSTM) architecture are an excellent method for exploiting the time-series-based sequential data structures of ECG signals. Using an LSTM architecture with a powerful technique in ECG signal delineation classifies the ECG waveform from one step to the next time step for each heartbeat [14]. However, the lack of feature extraction in LSTM makes the obtained results less optimal. This study proposes a deep structure of a CNNs as a feature extraction and LSTM as a classifier model to overcome this limitation. The convolutional layers automatically extract these ECG features, and LSTM classify the waveforms into four distinct component waveforms (i.e., P-wave, QRS complex, T-wave, and isoelectric line) in each heartbeat.

In most previous studies, the proposed delineation process is only for localizing and classifying the ECG rhythm without interpreting abnormalities. This study proposes a delineation process for identifying the rhythm abnormality of AF. To our knowledge, no previous study has investigated AF screening with a delineation approach. The important finding in our research is that we used a single model architecture to simultaneously predict the presence or absence of all labels and interpret the entire ECG waveform. The final post-processing step is reconciling and aggregating the information from the ECG waveforms and providing a comprehensive analysis of the signal to make an AF interpretation. Therefore, this study and the proposed method make the following new contributions:

- To propose a framework of automatic identification with a single-lead ECG-based delineation approach.
- To develop the stacked CNNs-Bi-LSTM architecture for ECG waveform delineation with a massive amount of data.
- To implement the combination of delineation approach and medical knowledge base learning to aggregate the information in an ECG signal simultaneously to detect the absence of P-wave, the ventricular response, and the irregularity of the RR interval.
- To evaluate the proposed model for automatic AF interpretation with clinical data from single-lead personal ECG devices.

With a detailed approach to the problem and to describe the proposed method and its contribution, this paper is structured as follows: In Section 2, we present materials and methods proposed for ECG delineation in identifying AF, while in Section 3, we present the results of applying the methods and a discussion. Finally, Section 4 draws conclusions from the process and the obtained results.

## 2. Methodology

**Figure 1:**
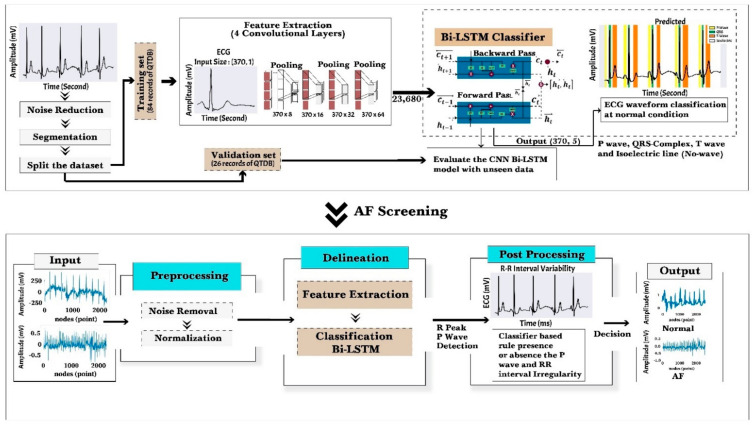
The proposed framework with ECG the delineation approach and AF identification. Automated ECG signal delineation is developed with convolutional layers as feature extraction and Bi-LSTM as a classifier.

This section describes our proposed methodology for AF identification, utilizing a delineation process. A sophisticated conceptual framework is proposed by incorporating various established processes to facilitate the ECG waveform delineation and identification of AF. Two blocks are represented in the framework: (i) ECG waveform signal delineation process and (ii) AF screening by interpreting ECG delineation result and medical knowledge representation. The ECG delineation and AF identification process pipeline consists of various stages, as shown in Figure 1. At the delineation baseline, we used an ECG signal in normal sinus rhythm (NSR) to classify four waveforms, i.e., P-wave, QRS complex, T-wave, and isoelectric line (no wave). The isoelectric line is the baseline of the ECG, recorded in the TP interval during rhythm with P-waves. The whole methodology is described in the next sub-section as follows.

### 2.1. Dataset Preparation

The proposed delineation method was validated on two well-known annotated databases QT Database (QTDB) [17] and a lead II ECG from The Lobachevsky University Database (LUDB) [18]. In comparison, AF identification utilized the PhysioNet/CinC Challenge 2017 database [19], The China Physiological Signal Challenge 2018 database [20], and ECG recordings from Mohammad Hoesin Indonesia General Hospital. The whole data distribution is seen in Table 1. 

### 2.2. Noise Removal

The baseline wander noise must be removed from the raw signal to produce satisfactory results from the delineation process. These factors were removed by applying a discrete wavelet transformation (DWT) with low-pass and high-pass filters [14,21]. This strategy can separate the ECG signal into various frequency bands and maintain a good representation of a nonstationary signal [7,22]. While each signal is decomposed per level to remove ECG noise, DWT produces half of the input signal frequency. This process was conducted in approximately eight stages of decomposition. The noise removal process was carried out using soft-thresholding techniques and universal-threshold rules for each signal decomposition. The decomposition results were reconstructed back into the initial signal cleaned of noise. 

### 2.3. Segmentation

Segmentation is a crucial stage in the delineation process for classifying the whole waveform pattern of the ECG signal. The process is conducted by dividing the short single-lead ECG signal into a beat-to-beat segment to achieve a proper window size. Segmentation started from the P-wave and went to the isoelectric line after the T-wave, which contained one heartbeat at an NSR heart rate. These segment points were sampled with around 250 Hz for three beats. However, it should be noted that the continuity of the time series (ECG signal) within each segment was preserved to make it workable for the network to learn the pattern of different waveforms from each input. After the beat-by-beat segmentation process, we have the data for the delineation process, as seen in Table 2.

### 2.4. CNN-Bi-LSTM

A previous study achieved best performance when the ECG signal was delineated automatically with a CNNs and bidirectional LSTM (Bi-LSTM) architecture [21]. A unidirectional phase also has limits because future input information is impossible to get from the current state. On the other hand, bidirectional does not necessitate correcting its input data. Furthermore, the current state allows access to future input data [23]. The feature extraction of CNNs process used four convolutional layers without max pooling. The convolution process can be expressed as follows [7,21]:

(1) $$a_{ij}^{m}=\varphi (b_{i}+\sum_{k=1}^{M}w_{ik}x_{j+k-1})=\varphi (b_{i}+w_{i}^{T}x_{j})$$

where $a_{ij}^{m}$ is the activation of the j neuron of the i filter for the m convolutional layer, M is the kernel size [8], φ is the neural activation function, b is the shared bias of the i filter, $w_{i}={\left[\begin{array}{ccc} w_{i1} & w_{i2} & \ldots \end{array}w_{iM}\right]}^{T}$ are the shared weights of the i filter, and $x_{j}=[\begin{array}{ccc} x_{j} & x_{j+1} & \ldots x_{j+M-1}{]}^{T} \end{array}$ are the corresponding M inputs. 

**Figure 2:**
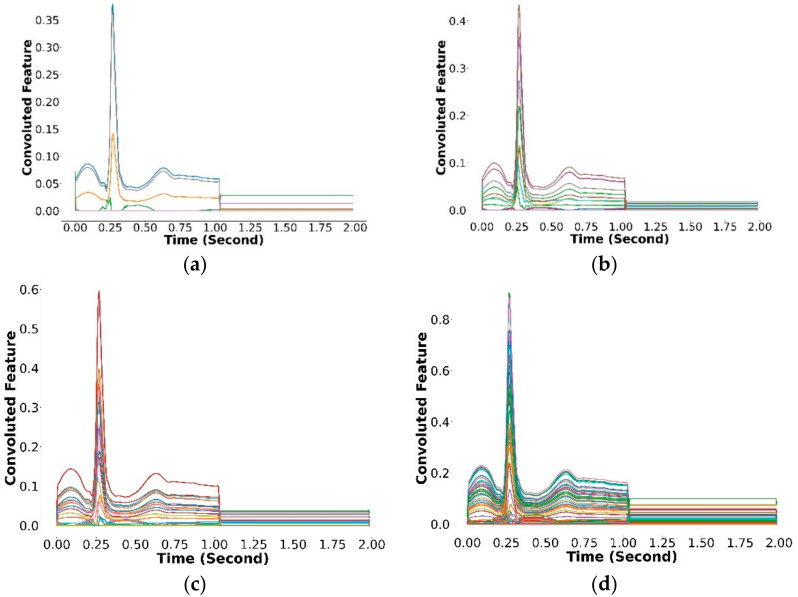
The result of the feature map from convolutional layers. (**a**) Convolution 1 (370, 8). (**b**) Convolution 2 (370, 16). (**c**) Convolution 3 (370, 32). (**d**) Convolution 4 (370, 64).

In this study, the convolutional layer received two input values: the ECG beats and corresponding labels, including P-wave, QRS complex, T-wave, isoelectric line (no wave), and zero padding. The ECG beat had a size of (370, 1), which contains information about the class type of each ECG node. We divided the classes into P-wave as class 0, QRS complex as class 1, T-wave as class 2, the isoelectric line (no wave) as class 3, and zero padding as class 4. The network was combined with the rectified linear unit (ReLU) activation function. This function has been adopted with four convolution layers (8, 16, 32, and 64 filters), resulting in an output size (370, 64). This output size means that the features were set into 370 timesteps, containing 64 features per timestep. The output from the convolutional part was adjusted to feed into the Bi-LSTM part as a classifier. We used the *OneHotEncoder*() function to encode categorical features as a one-hot numeric array, resulting in a shape of (370, 5). The one-hot array was then fed into the Bi-LSTM classifier model as a target along with the ECG waveform as an input. The feature map resulting from the convolutional layers is shown in Figure 2. 

The proposed model is to classify the positions and magnitudes of the following durations, such as P-wave, QRS complex, and T-wave. The Bi-LSTM architecture processes a data sequence in both directions by two hidden layers, which are fed forward to the output layer and backward along with their corresponding weights (W) and biases (b). Two parallel directions of forwarding and backward passes are provided below [21].

(2) $$a^{t}=\tanh(W_{c}x^{t}+U_{c}h^{t-1})$$

(3) $$i^{t}=\sigma (W_{i}x^{t}+U_{i}h^{t-1})=\sigma ({\overset{⌢}{i}}^{t})$$

(4) $$f^{t}=\sigma (W_{f}x^{t}+U_{f}h^{t-1})=\sigma ({\overset{⌢}{f}}^{t})$$

(5) $$o^{t}=\sigma (W_{o}x^{t}+U_{o}h^{t-1})=\sigma ({\overset{⌢}{o}}^{t})$$

(6) $$LST{\vec{M}}_{ft}^{1}=\tanh(W_{i\vec{h}}^{1}x_{t}+W_{\vec{h}\vec{h}}^{1}LSTM_{t-1}^{\vec{1}}+b_{\vec{h}}^{1})$$

(7) $$LST{\overset{\leftarrow }{M}}_{bt}^{1}=\tanh(W_{i\overset{\leftarrow }{h}}^{1}x_{t}+W_{\overset{\leftarrow }{h}\overset{\leftarrow }{h}}^{1}LSTM_{t+1}^{\overset{\leftarrow }{1}}+b_{\overset{\leftarrow }{h}}^{1})$$

From Equations (6) and (7), the output of the Bi-LSTM layer at a time t:

(8) $$y_{t}^{1}=\tanh(W_{h\vec{o}}^{1}LST{\vec{M}}_{t}^{1}+W_{h\overset{\leftarrow }{o}}^{1}LST{\overset{\leftarrow }{M}}_{t}^{1}+b_{0}^{1})$$

where $a^{t}$ is input, $i^{t}$ is input gate, $f^{t}$ is forget gate, $o^{t}$ is output gate, h is hidden gate, the output depends on ${\vec{LSTM}}_{t}$ and $L{\overset{\leftarrow }{STM}}_{t}$; $h_{0}$ is initialized as a zero vector.

In the process of ECG waveform classification, a fixed window size of 370 nodes for each sequence was used. The window size was large enough to capture one heartbeat (from the start of P-wave 1 to the start of P-wave 2). The total of the nodes’ starts-to-end index of the P-wave, QRS complex, T-wave, and the isoelectric line had to be adjusted in the unit of seconds, respectively. These duration points were sampled with a sampling frequency of about 250 Hz. The classifier architecture used in this study consisted of an input layer size (370, 1) with five output layers, producing 512 hidden layer neurons, including the Tanh–Sigmoid hidden activation function. The output layer activation function was Softmax, with a learning rate of 0.0001, and the epoch was 300. The network optimizer utilized was the Adam optimizer, and categorical cross-entropy was selected as a loss function to measure the loss value of the classifier model. The output from this classifier contains the probability class for each node. One class with the highest probability value was selected as the predicted output of the model. In the end, a vector with a size of (370, 1) was formed as the predicted class of each node.

The entire structure of the feature extraction and classification process by the CNNs-Bi-LSTM architecture is shown in Table 3.

### 2.5. Model Delineation Evaluation

The performance of the proposed Bi-LSTM model was validated using a confusion matrix. To evaluate classification performance, we applied performance metrics [24,25]. Furthermore, we used a positive predictive value (precision) versus a sensitivity (recall) or precision–recall (P–R) curve to measure all potential cut-offs in the delineation process. The higher area under the curve (AUC) value was the P-R curve [24]. For model configuration and evaluation, we conducted the experiments on a workstation with one Intel(R) Core (TM) i7-10700K CPU, with a 3.80 GHz processor and 32 GB RAM, and one NVIDIA GeForce RTX 2070 Super 8 GB GPU. All experiments were run on Windows 10 Pro 64-Bit and CuDNNLSTM.

### 2.6. AF Identification

**Figure 3:**
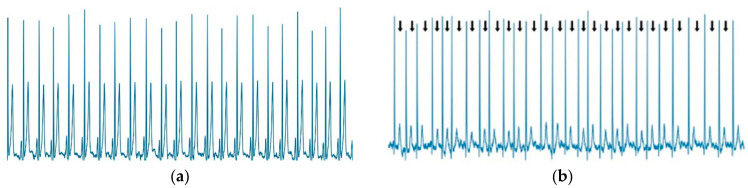
Example of ECG signal: (**a**) normal rhythm as NSR and (**b**) abnormal rhythm as AF.

In measuring the diagnostic performance of AF, it is common to leverage an ECG, a signal that measures the heart’s electrical activity. As shown in Figure 3, a special hallmark of AF in the absence of P waves is that the rhythm is irregularly irregular, and the morphology of the P-waves is fibrillation [24]. This study conducted two important stages for making an automatic AF interpretation from the ECG signal: detecting the heart-rate variability (HRV) to measure the regular or irregular condition and detecting the presence or absence of P-waves in the ECG record to support AF interpretation.

An AF identification procedure after ECG signal delineation is as follows:

- Each ECG device has a different frequency sampling and to produce the generalization model of delineation, to determine one beat, it was segmented again around 0.2 s before the $R_{peak}$ and 0.45 s after the $R_{peak}$.
- From the delineation result, we can determine the occurrence P-wave pattern; however, the QRS complex should be processed to determine heart-rate irregularity. To measure the distance between $R_{peak}$-$R_{peak}$ or RR, intervals were calculated using the following equation:
  (9) $$RR^{Interval}=(R_{peak}[i]-R_{peak}[i+1])*fs*1000$$
  where $R_{peak}[i]$ is $R_{peak}$ position at *i* node, $R_{peak}[i+1]$ is $R_{peak}$ position at (*i* + 1) in nodes, *fs* is the frequency sampling, and RR interval values in beat per minutes (BPM).
- From the RR interval result, the ECG signal was checked to determine whether there was a continuous change in ventricular response in five to seven beats to indicate whether the ECG has a regular or an irregular rhythm in the ECG recording.
- The three BPM ranges of ventricular response are needed to ensure whether a signal is normal or AF condition, as (i) the normal ventricular response group has the RR interval value ranged between 60 and 100 BPM; (ii) the slow ventricular response group has the RR interval value less than 60 BPM (<60 BPM); and (iii) the rapid ventricular response group has the RR interval greater than 100 BPM (>100 BPM).
- After determining the ventricular response of the ECG signal, then the regular or irregular rhythm is determined. An ECG signal is said to be a Regular Rhythm if that rhythm has a pattern. The rhythm pattern can be a normal, rapid, or slow ventricular response. On the other hand, if there is no pattern occurred in the ECG signal, then the signal is categorized as an irregular rhythm.
- By using two inputs, the AF or NSR was interpreted according to the following rules:
  - If the P-wave was present and the rhythm regular, then the condition was normal.
  - If the P-wave was absent and the rhythm regular, then the condition was normal.
  - If the P-wave was present and the rhythm irregular, the condition was AF.
  - If the P-wave was absent and the rhythm irregular, the condition was AF.

## 3. Results and Discussion

Morphology in the ECG signal can be represented by the cardiac waveform series of the P-wave, the QRS complex, the T-wave, and the isoelectric line (no wave). This study has proposed a training, validation, and testing set for the delineation task. The validation set is used for the hyperparameter tuning, while the testing set as unseen data is used to show the generalization abilities of the CNNs-Bi-LSTM model.

This study classified such a waveform series using 90% for the training process and 10% for the testing process with shuffle sampling. The total training set was 7714 samples, and the testing set was 858 samples. The CNNs-Bi-LSTM result was compared to the data annotation by *ecgpuwave* in the QTDB, and we also compared the LSTM and Bi-LSTM classifiers without feature extraction. The *ecgpuwave* provided the exact location of all waveforms in the signal. The *ecgpuwave* output was written as a standard WFDB-format annotation file related to the specified annotator that was utilized as label or ‘ground truth’ for ECG waveforms.

### 3.1. Model Evaluation of the Delineation Process

**Figure 4:**
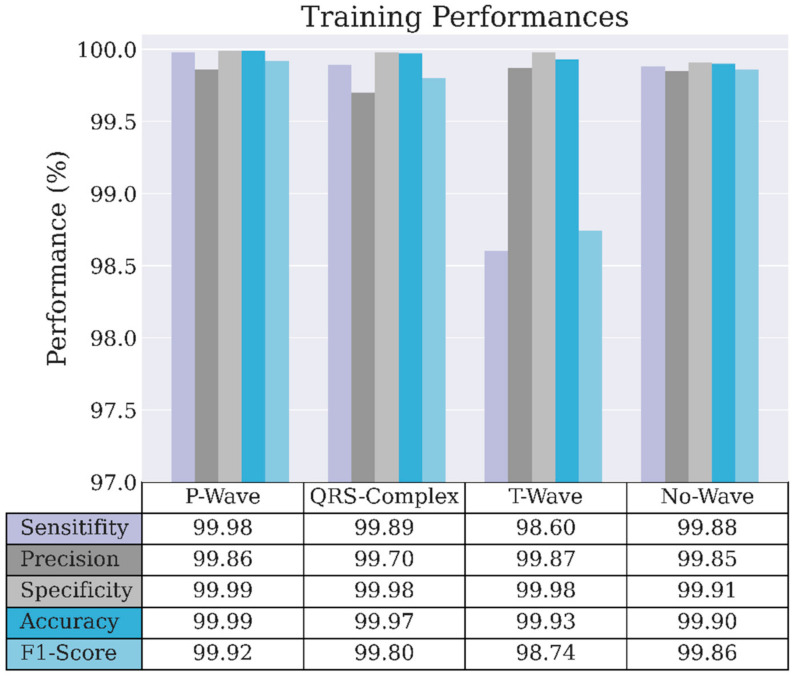
Model evaluation for the training process in normal ECG waveform.

**Figure 5:**
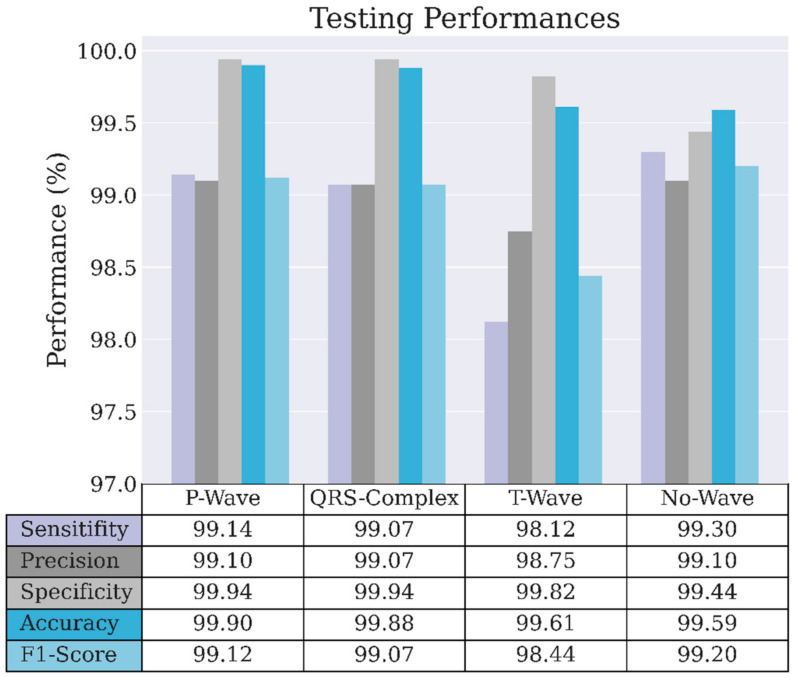
Model evaluation for the validation process in normal ECG waveform.

The LSTM standard has a unidirectional phase that preserves the past information and runs the inputs only in forward passes. In contrast, the Bi-LSTM phase runs inputs in forward and backward passes and preserves the information from both the past and future. Figure 4 and Figure 5 show the performance of the proposed CNNs-Bi-LSTM classifier in the training and validation evaluation. All classification performances reached over 99% in the training phase. Only the T-wave classification produced 98.75% precision, 98.12% sensitivity, and a 98.44% F1-score in the validation phase, while the remaining achieved over 99%.

Three scenarios were created to validate the proposed model with an intra–inter patient in normal rhythm and arrhythmia conditions. Most previously reported works on medical data in the literature have been evaluated only for the intra-patient paradigm rather than the inter-patient scheme, which is a more realistic scenario to prevent training and to test the model using samples from the same patients. Therefore, some of these methods achieved good accuracy using the intra-patient scheme; however, their results are unreliable, as their evaluation process was biased. In addition, the input data has never been recognized in the training process in the testing processes that use inter-patient data. In some cases, it can cause the performance results to decrease significantly.

The waveform classification performance of the three scenarios is shown in Table 4. By using inter-patient data, the CNNs-Bi-LSTM model has maintained classification performance. However, in the inter-patient (NSR) scenario, the sensitivity decreased by about 9%, and the precision decreased by about 4%. Such conditions happened due to the inter-patient data having variations in signal morphology, and the noise suppressed the ECG signal. We also tested the delineate system with abnormal data in the arrhythmia condition (ventricular premature complexes and atrial premature complexes). Ventricular and atrial premature complexes occur when the lower chambers of the heart contract before they should. When this happens, the heartbeat becomes out of sync. This can produce a regular heartbeat, an extra heartbeat, a pause, and a stronger heartbeat. With such an arrhythmia condition, our model delineation performance decreased 1.47% in sensitivity, 1.48% in precision, 0.28% in specificity, and 0.48% in accuracy (see Table 4). However, our model still maintained performance over 97% in sensitivity, which means that the model produces a high true positive rate value.

For another experiment with the same hyperparameters, the ECG signals of the LUDB were implemented in an intra-patient scenario to analyze the performance comparison of the delineation algorithm. All the LUDB recordings were validated under various conditions, such as sinus rhythm, sinus tachycardia, sinus bradycardia, sinus arrhythmia, irregular sinus rhythm, AF, and atrial flutter. With the massive data of the LUDB, the performance of the delineation algorithm was applied in various conditions, and all performances were achieved above 95.61%. Thus, the proposed CNNs-Bi-LSTM model was still robust for the delineation task, and there was no significant gap between the QTDB and the LUDB in the intra-patient scenario (see Table 4). The performance decrease witnessed by using the LUDB could be due to various signal abnormalities, such as Sinus Rhythm, Sinus Tachycardia, Sinus Bradycardia, Sinus Arrhythmia, Irregular Sinus Rhythm, AF, and Atrial Flutter. The enormous difference in recorded numbers could be more challenging. The LUDB had 200 records, while the QTDB used only 10 and 15 records for NSR and arrhythmia, respectively. Hence, the LUDB offers a variety of complex morphologies, which can be a challenge in the delineation process.

**Figure 6:**
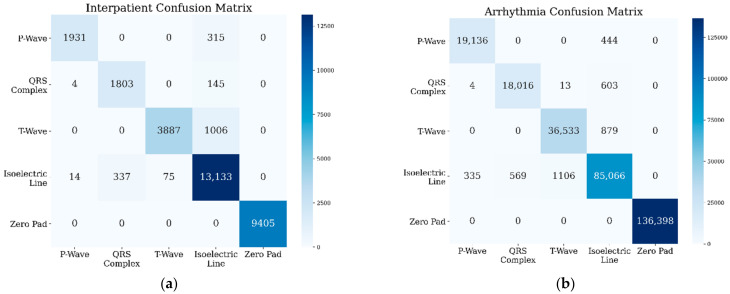

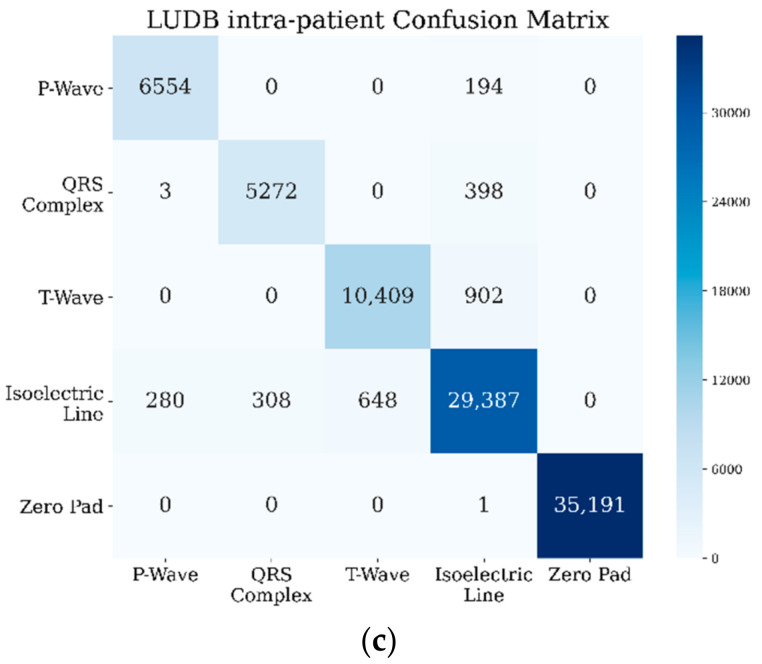
Confusion matrices for QTDB’s (**a**) inter-patient and (**b**) arrhythmia conditions, and (**c**) LUDB’s intra-patient.

For QTDB, the confusion matrices for NSR with inter-patient cases showed that the P-wave class was misclassified in about 315 samples, the QRS complex in about 145 samples, and the T-wave in about 1006 samples (refer to Figure 6a). This meant that the nodes shifted from all samples to the isoelectric line; however, no sample was misclassified into another waveform class. When testing for the arrhythmia condition, as shown in Figure 6b, the QRS complex class was misclassified as another waveform class, three samples were misclassified as P-waves, and 13 samples were misclassified as T-waves. However, due to the large number of ECG recordings in the MIT-BIH Arrhythmia dataset, the waveform classification results in terms of their sensitivity and precision performances still reached over 97% (see Table 4). The shift of the waveform class to the isoelectric line class occurred in three classes: P-wave, QRS complex, and T-wave. On the other hand, when the model was validated in LUDB, P-wave, QRS-complex, and T-wave were misclassified as isoelectric lines (refer to Figure 6c). 

**Figure 7:**
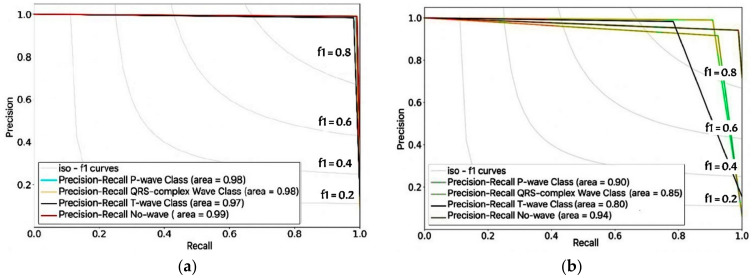
Model evaluation based on precision–recall (P–R) curves. (**a**) intra-patient data. (**b**) inter-patient data.

Figure 7a,b shows another metric of delineation performance in the intra- and inter-patient scenarios: the precision–recall (P–R) curve to evaluate experimental performance. Precision can be seen as quality, whereas recall can be seen as a measure of quantity. The P-R curve was essential in this study because the ECG delineation had four classes of waveforms and zero padding along with the signal. Therefore, the shift in each class duration will affect the delineation process’s precision performance.

The P-R curve was utilized to show the false positive and false negative rate values. The two curves show good results utilizing CNNs-Bi-LSTM models. The intra-patient data produces an average P-R curve area of 1.0 and the inter-patient data, producing an area of 0.98, which shifts to the upper right. The high area under the curve (AUC) represents both high recall and precision, where high precision relates to a low false-positive rate and high recall relates to a low false-negative rate. In the intra-patient data, all classes produced an AUC of over 97%. However, in the inter-patient data, the AUC of the QRS complex and T-wave reached 85% and 80%, respectively. This means that the duration of the two waves shifted by 13–17% to the isoelectric line or no-wave class. There were no positive or negative electrical charges to create deflections. 

**Figure 8:**
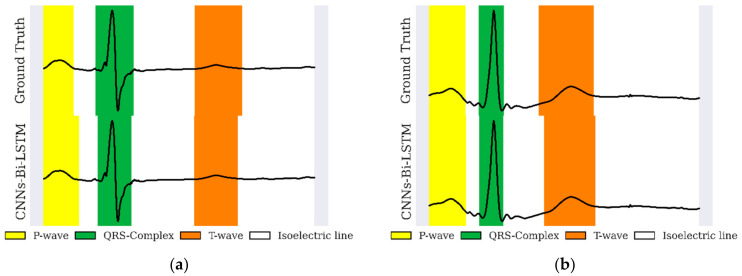
A sample of one-beat ECG signal delineation results for (**a**) intra-patient and (**b**) inter-patient in NSR data. Each figure represents the QTDB ground truth (top figure) and CNNs-Bi-LSTM (bottom figure).

Figure 8 depicts a sample delineation result of one beat in the NSR with intra–inter-patient data for QTDB. The result appeared to indicate a satisfactory waveform pattern; however, in the long ECG signal recording, the wave’s duration shifted to the isoelectric line in a large amount of inter-patient data. This must be improved significantly to produce exact waveform delineation results (see Table 4). 

**Figure 9:**
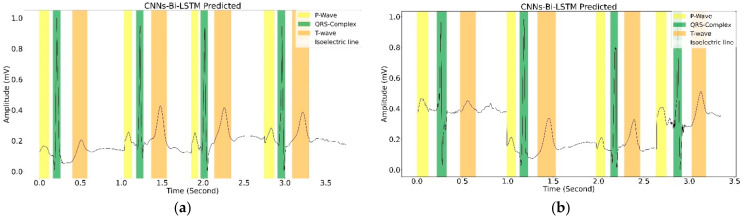
The four-beat sample of ECG-signal delineation in (**a**) NSR and (**b**) arrhythmia.

This work presents a CNNs-Bi-LSTM-based approach for ECG delineation by framing the problem as a classification task. Our work exhibits good delineation performance, and the network has excellent classification performance in single-lead scenarios with unseen data (inter-patient), as depicted in Figure 9. Our approach is data-driven. Therefore, any bias in the QTDB will be learned by the network. In this sense, CNNs-Bi-LSTM-based approaches, such as the proposed model, can easily assimilate newly annotated data to enhance delineation performance. Development became shortened once the right design decisions had been modeled, thus becoming an alternative to DL-based methods with great potential. In Figure 9, we plotted the sample of delineation results in four beats. The classification results showed satisfactory performance. CNNs-Bi-LSTM can recognize several ECG morphologies in terms of P-wave, QRS complex, and T-wave, along with a signal recording. 

We compared the proposed delineation process with two other networks: unidirectional (Uni-LSTM) and Bi-LSTM architectures without CNNs. The feature extraction performed by convolutional layers can improve the proposed model’s classification performance (see Table 5). The average precision classification of the waveform increased from 98.97% to 99.01% using the proposed model, and the other metrics also increased. In addition, we compared our proposed model to other deep learning algorithms for the delineation task (see Table 6). Based on the results for the P-wave, QRS complex, and T-wave detection, the performance in our delineation model significantly outperformed the other methods proposed by [14,25] and [15] due to all their methods reaching metrics under 98%.

Furthermore, while the performance results obtained by [26,27] were excellent (around 99%), their detection focused only on the QRS complex. Our study generated the delineation algorithm for the P-wave, QRS complex, T-wave, and isoelectric line (no wave). The three main deflections of ECG waveforms are essential for practical diagnosis. The P-wave is caused by the depolarization wave spreading over both atria. The QRS complex is written when the depolarization wave reaches the ventricles. A period of electrical inactivity follows, during which the ECG records the ST segment. Finally, the ventricular muscle slowly recharges in preparation for the next cardiac beat, and this repolarization of the ventricles appears as a T-wave. Thus, it can be concluded that our proposed model had a good result in the delineation process in the inter-patient data of the QTDB, with all performances achieving above 98.91% (see Table 6).

### 3.2. AF Identification Results

We conducted experiments for AF identification with three datasets taken from (i) the China Physiological Signal Challenge 2018, (ii) the PhysioNet Computing in Cardiology Challenge 2017, and (iii) the Mohammad Hoesin General Hospital Indonesia. The three datasets have different sampling frequencies in their ECG recordings. Meanwhile, the design delineation model uses the NSR QTDB with a sampling frequency of 250 Hz. The difference between the data model and the data tested will be challenging because it will affect the nodes generated in each beat. AF can be identified by confirming three decision-making factors: the P-wave’s absence in duration 0.06–0.08 s and considerably higher beats per minute than 60–100 BPM with inconsistent RR interval variations and not within the 0.6–1.0 s range [27]. The identification results were evaluated based on positive predictive value (PPV) or the probability that subjects with a positive screening test truly have the disease. A negative predictive value (NPV) is the probability that subjects with a negative screening test truly do not have the disease. The AF identification results can be summarized as follows:

- The China Physiological Signal Challenge 2018 database was collected from 11 hospitals and contained 12-lead ECG recordings lasting 6–60 s, this study used only lead II. In the identification process, we used about 826 NSR records and 988 AF records. The results showed that we had 95.40% PPV, 84.55% NPV, and an 89.65% F1-score. There were 38 (4.6%) records that had a false negative (FN), and 144 (14.5%) records that had a false positive (FP).
- The PhysioNet Computing in Cardiology Challenge 2017 database was collected from a short single-lead ECG recording between 30 and 60 s in length. ECG recordings were collected using a mobile Cardia with the AliveCor device. We utilized about 3154 NSR records and 771 AF records. The experiment reached 86.51% PPV, 95.95% NPV, and an 90.43% F1-score. There were 457 (14.4%) records with FN and 114 (1.4%) records with FP.
- The General Mohammad Hossein Hospital (Palembang, Indonesia) database was collected from a short single-lead ECG recording between 30 and 60 s in length. ECG recordings were collected using a mobile Cardia with the AliveCor device. We utilized about 78 NSR records and 45 AF records. The experiment reached 85.61% PPV, 97.10% NPV, and a 90.41% F1-score. There were 12 (15.3%) records with FN and two (4.4%) records with FP.

A short single-lead ECG demonstrated an overall greater NPV or sensitivity value from the experiment. This means that the proposed model makes it possible to identify cardiac abnormalities from a single-lead ECG, even when the recordings are of short duration, with delineation and medical knowledge representation. One of the questions this work has addressed is the clinical impact that this method can achieve. Thus, it was critical to evaluate the model in a real dataset known for its many patients and signals. Even though we are critical of some of the manual annotations in the QTDB from PhysioNet, we assume that most of the annotations are correct and can be used for evaluation. The main limitation of this work is the lack of more up-to-date databases that contain a higher variability for a more comprehensive array of pathologies. Despite ECG usually being the first information registered of a patient’s cardiac condition, not many large, annotated databases for ECG delineation exist.

## 4. Conclusions

This research aimed to develop a non-invasive method to detect AF conditions using a single-lead ECG signal delineation approach. Although we did not test the proposed model with clinical data, the model was validated and tested with massive data from three datasets. Even though this identification system is not a direct replacement as a standard ECG, the utilization of this affordable system will contribute toward reducing strokes, raising heart health awareness, and significantly saving on costs. The AF identification system will allow physicians with less knowledge of how to evaluate ECG signals or other related factors to get a simple identification of potential AF concerns. The proposed CNNs-Bi-LSTM network demonstrated significant precision in identifying AF with a specific algorithm, and it can be improved as a generalized model for other heart diseases. In the future, further research will focus on how to better integrate physicians’ experience and expertise into the DL model to improve the performance of the ECG delineation task, and to generalize our approach to cover more cross-disciplinary work.

## Figures and Tables

**Table 1:** The data distribution for the delineation and identification process.

Database	Records	Description	Frequency Sampling
Delineation Model			
QTDB	10 subjects NSR	Training/validation	250–500 Hz
	15 subjects Arrhythmias	Training/validation	
	Two subjects NSR	Testing	
LUDB	200 subjects for NSR, tachycardia, bradycardia, arrhythmia, irregular rhythm, AF, atrial flutter	Other data set for model validated	500 Hz
Medical knowledge-based learning			
PhysioNet/CinC Challenge 2017 database	5154 subjects NSR	AF identification	300 Hz
	771 subjects AF		
The China Physiological Signal Challenge 2018 database	918 subjects NSR	AF identification	500 Hz
	1098 subjects AF		
ECG recordings from Mohammad Hoesin Indonesian Hospital.	78 subjects NSR	AF identification	300 Hz

**Table 2:** The ECG beat segmentation to develop the delineation model.

Data	Training (Beats)	Validation (Beats)	Testing (Beats)
QTDB	14,376	1639	100
LUDB	1096	122	-

**Table 3:** The structure of the proposed CNNs-Bi-LSTM architecture.

Layer	Input Nodes	Filter Number	Kernel Size/Pool Size	Output Nodes	Feature Interpretation
Input	370, 1	-	-	-	ECG amplitude for one beat
Convolution 1	370 × 1	8	3 × 1, stride 1	370 × 8	8 feature maps
Convolution 2	370 × 8	16	3 × 1, stride 1	370 × 16	16 feature maps
Convolution 3	370 × 16	32	3 × 1, stride 1	370 × 32	32 feature maps
Convolution 4	370 × 32	64	3 × 1, stride 1	370 × 64	64 feature maps
Bi-LSTM	370 × 64	-	-	370 × 1024	Two directions of feature data (512 nodes for both forward and backward directions)
Output	-	-		370 × 5	370 nodes with five classes (P-wave QRS-complex, T-wave, No-wave, and zero padding)

**Table 4:** Delineation results in an intra–inter patient with NSR, arrhythmia, and various conditions using the QTDB and the LUDB.

Database	Records	Scenario	Classification Performance (%)				
			Sen.	Prec.	Spec.	Acc.	F1-Score
QTDB	10	Intra-patient (NSR)	98.91	99.01	99.79	99.79	98.96
	15	Intra-patient (Arrythmia)	97.44	97.53	99.57	99.31	97.48
	2	Inter-patient (NSR)	89.90	94.30	97.86	97.33	91.70
LUDB	200	Intra-patient (Various Conditions)	95.61	95.93	99.18	98.77	95.76

Note: Sensitivity (Sen.), Precision (Prec.), Specificity (Spec.), Accuracy (Acc.).

**Table 5:** The three LSTM architectures with the best result.

Architecture	Performance (%)				
	Sen.	Prec.	Spec.	Acc.	F1-Score
Uni-LSTM	98.71	98.80	99.75	99.64	98.75
Bi-LSTM	98.84	98.97	99.68	99.68	98.91
Convolutional-Bi-LSTM	98.91	99.01	99.79	99.79	98.96

Note: Sensitivity (Sen.), Precision (Prec.), Specificity (Spec.), Accuracy (Acc.).

**Table 6:** Comparison with other delineation using deep learning model with the same QTDB records.

Architecture	Detection	Performance (%)				
		Sen.	Prec.	Spec.	Acc.	F1-Score
Convolutional LSTM [7]	P-wave, QRS-complex, T-wave, and No wave	97.95	95.68	-	-	96.78
Convolutional Neural Network-UNet [25]	P-wave, QRS-complex, and T-wave	99.51	95.83	-	-	-
Convolutional Long Short-Term Memory [15]	P-wave, QRS-complex, and T-wave	94.47	94.19	-	94.75	94.66
Convolutional Neural Network [26]	QRS-complex	99.97	99.99	-	-	99.98
Convolutional Neural Network [27]	QRS-complex	99.10	99.00	-	-	-
Proposed model	P-wave, QRS-complex, T-wave, and Isoelectric line (no-wave)	98.91	99.01	99.79	99.79	98.96

Note: Sensitivity (Sen.), Precision (Prec.), Specificity (Spec.), Accuracy (Acc.).

## Data Availability

Not applicable.
